# Structural Insights into Substrate Specificity of Feruloyl-CoA 6’-Hydroxylase from *Arabidopsis thaliana*

**DOI:** 10.1038/srep10355

**Published:** 2015-05-20

**Authors:** Xinxiao Sun, Dayong Zhou, Palani Kandavelu, Hua Zhang, Qipeng Yuan, Bi-Cheng Wang, John Rose, Yajun Yan

**Affiliations:** 1 State Key Laboratory of Chemical Resource Engineering, Beijing University of Chemical Technology, Beijing 100029, China; 2 Department of Biochemistry & Molecular Biology, University of Georgia, Athens, Georgia 30602, USA; 3 BioChemical Engineering Program, College of Engineering, University of Georgia, Athens, Georgia 30602, USA

## Abstract

Coumarins belong to an important class of plant secondary metabolites. Feruloyl-CoA 6’-hydroxylase (F6’H), a 2-oxoglutarate dependent dioxygenase (2OGD), catalyzes a pivotal step in the biosynthesis of a simple coumarin scopoletin. In this study, we determined the 3-dimensional structure of the F6’H1 apo enzyme by X-ray crystallography. It is the first reported structure of a 2OGD enzyme involved in coumarin biosynthesis and closely resembles the structure of *Arabidopsis thaliana* anthocyanidin synthase. To better understand the mechanism of enzyme catalysis and substrate specificity, we also generated a homology model of a related ortho-hydroxylase (C2’H) from sweet potato. By comparing these two structures, we targeted two amino acid residues and verified their roles in substrate binding and specificity by site-directed mutagenesis.

Coumarins (1, 2-benzopyrones) are a major group of plant secondary metabolites. They play important roles in the environmental adaptation of plants and contribute to the defense against phytopathogens^1^,^2^. Coumarin derivatives have demonstrated multiple pharmaceutical activities such as anticoagulative, antibacterial, anti-inflammatory, etc^3^,^4^,^5^. For instance, 4-hydroxycoumarin is the synthetic precursor of warfarin, which is among the most widely used anticoagulant pharmaceuticals for the treatment of thromboembolic disorders^4^. In addition, coumarins have also shown anti-HIV and anti-tumor activities^6^,^7^.

In plants, coumarins are synthesized via the general phenylpropanoid pathway^8^. A key step in the formation of coumarin is the ortho-hydroxylation of the aromatic ring of cinnamic acid. Studies showed that this enzyme activity is located within the chloroplast fraction from *Melilotus alba*^9^. Recently, the actual enzyme involved was identified and characterized from *A. thaliana* and was designated as feruloyl-CoA 6’-hydroxylase (F6’H1)^10^. The product 6’-hydroxyferuloyl-CoA is converted into a simple coumarin scopoletin via spontaneous *trans/cis* isomerization and lactonization (Fig. 1). This enzyme belongs to 2-oxoglutarate dependent dioxygenase (2OGD) family^10^. Members of this enzyme superfamily catalyze an amazing variety of reactions, including protein side-chain modifications, lipids metabolism, alkylated DNA/RNA repair, biosynthesis of antibiotics, and plant metabolites^11^. Most members of the family couple the oxidative decomposition of 2-oxoglutarate (forming succinate and CO_2_) to the hydroxylation of a co-substrate^11^.

F6’H1 consists of 361 amino acid residues and shows significant homology to other plant 2OGDs such as anthocyanidin synthase from *A. thaliana* (34% identity), flavanone 3b-hydroxylase from *P. hybrida* (34% identity), gibberellin 3b-hydroxylase from *Pisum sativum* (32% identity). Sequence alignment showed that F6’H1 contains the conserved Fe(II)-binding motif (His-X-Asp-Xn-His) and the 2-oxoglutarate C5 carboxy group binding motif (Arg-X-Ser). The activity of F6’H1 is entirely dependent on the presence of 2-oxoglutarate and the Fe (II) ion. Feruloyl-CoA was the optimal substrate for F6’H1. F6’H1 only shows trace activity towards 4-coumaroyl-CoA and no activity towards ferulic acid^10^. Based on bioinformatic analysis, homologous enzymes were identified from other plant species, including *Ipomoea batatas (L.) Lam*, and *Ruta graveolens L.*^12^,^13^. However, compared with F6’H1, some of the enzymes showed distinct substrate selectivity. For example, C2’H, an ortho-hydroxylase from sweet potato had nearly equal activity towards feruloyl-CoA and 4-coumaroyl-CoA^13^. F6’H1 and C2’H have been successfully integrated into artificial pathways for the *de novo* production of scopoletin and umbelliferone in *E. coli*^14^.

In this study, we determined the crystal structure of F6’H1 apo enzyme by molecular replacement and also generated a homology model of C2’H structure. By comparison of two protein structures, we targeted two amino acid residues and verified their roles in enzyme activity and substrate selectivity by site-directed mutagenesis.

## Results and Discussion

Diffraction analysis of the colorless plate shaped F6’H1 crystals indicated that they belonged to space group C2, with unit-cell parameters a = 193.22 Å b = 54.55 Å, c = 78.82 Å and γ = 111.5°. Based on these unit cell dimensions and assuming two molecules per crystallographic asymmetric unit, the calculated Matthews coefficient is 2.38 Å^3^ Da^−1^ giving an estimated solvent content of ~48.2%^15^.

### The Overall F6’H1 Structure

**Figure 2:**
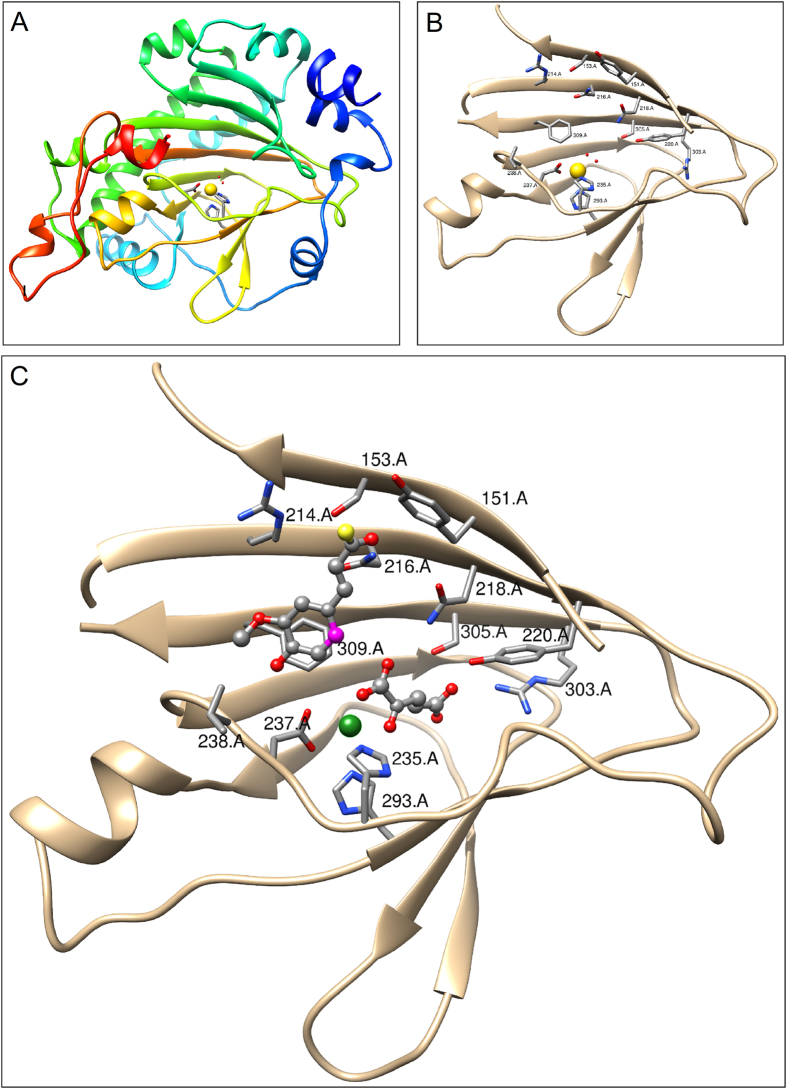
(**A**) A ribbon drawing of F6’H1 crystal structure (chain A) colored blue to red based on sequence position (N-terminal residues depicted in blue and C-terminal residues depicted in red). The sodium ion occupying the iron-binding site is colored yellow. (**B**) A ribbon drawing of the F6’H1 2OG-FeII_OXY domain. Residues involved in interactions with active site components are shown. Note the two water molecules interacting with the bound sodium ion that are mimicking 2OG binding to the catalytic iron. (**C**) A ribbon drawing of a theoretical model of the F6’H1 2OG-FeII_OXY domain showing active site components. The catalytic iron is colored green. The feruloyl group of the feruloyl-CoA substrate (upper left) and 2OG (lower right) are depicted using ball and stick representations. Position C6 of the feruloyl group, the site of enzymatic attack is highlighted in magenta. Note: the side chains of ASP 237 and ARG 303 have been adjusted from their positions in the F6’H1 structure to make close contact.

The F6’H1 structure (Fig. 2A) consists of residues A15-A343, B15-B141, B144- B344, 28 solvent molecules modeled as water and two sodium ions. The histidine purification tags, residues A1-A14, A344-A361 and residues B1-B14, B142-B143, B345- B361 were not observed in the electron density maps and are presumed to be disordered. As expected the F6’H1 structure closely resembles the structure of the *A. thaliana* Anthocyanidin synthase (ANS) search model. The two structures share a beta sandwich topology, and can be superimposed with an RMSD of superposition (242 α carbons) of 1.363 Å^16^. Like other members of this class of enzymes^17^,^18^,^19^,^20^,^21^ the structure contains an N-terminal DIOX_N (PF14226) domain, residues 62-172 linked to a C-terminal 2OG-FeII_OXY (PF03171) domain, residues 212 – 312 that contains the catalytic site^22^. Major features of the structure are the 15 helices and 14 β strands (Table 1). Strands β1, β2, β10, β7, β12, β5, β4 and β3 form an 8-stranded mixed β sheet (sheet S1), which assumes a β jellyroll fold common to this family of enzymes. Strands β6, β11, β8 and β9 forms an antiparallel β sheet (sheet S2), while strands β13 and β14 forms antiparallel β sheet (sheet S3). Sheets S1 and S2 together form a large (2,309 Å^3^) hydrophobic pocket that contains the catalytic site^23^. There are two enzyme molecules in the crystallographic asymmetric unit. A superposition of the two chains gives an RMSD of superposition of 0.670 Å for 320 Cα pairs^16^ with the largest deviations observed in the region containing α10 and spans the C-terminus of α9 to the N-terminus of strand S1β6.

### The Substrate Binding Pocket

The F6’H1 substrate binding pocket is contained within the C-terminal 2OG-FeII_OXY domain with its dominate feature being the 2-HIS-1-carboxylate facial triad (residues HIS 235, ASP 237 and HIS 293) that is involved in iron binding (Fig. 2B). However, in the apo structure reported here the iron site is occupied by a sodium ion. Strands S1β3 – S1β7 forms the back of the binding pocket. The catalytic triaid is positioned at the front of the binding pocket facing sheet S1 with HIS 235 and ASP 237 located in the long meandering loop connecting strands S1β4 to S1β6, and HIS 293 located at the N-terminal of strand S2β1.

The binding pocket is similar to that observed for the ANS search model giving an RMSD of superposition of 0.838 Å for 97 of the 113 Cα’s comprising strand S1β7 and the 2OG-FeII_OXY domain. Figure 2C shows a theoretical model^16^ of the F6’H1 active site based on the *A. thaliana* ANS crystal structure (PDB entries 1GP5, 1GP5 and 1GP6)^24^. Many of the key residues (ASN 218, TYR 220, ARG 303 and SER 305) involved in binding the 2OG co-substrate are structurally conserved in the F6’H1 structure. Interactions of the side chains of ARG 303, SER 305 and TYR 220 were used to anchor 5-carboxylate terminal of 2OG. This process required only slight rearrangements of the side chains involved. Interactions of the 2-keto and 1-carboxylate groups of 2OG with the sodium ion occupying the iron-binding site were used to anchor the other end of the molecule. The ferulic acid fragment of the feruloyl-CoA substrate was modeled using the ferulic acid molecule from PDB entry1JT2^25^ with O2 of the carboxyl group replaced by sulfur reflecting the CoA linkage. The ferulic acid fragment was placed using the (2R,3R)-trans-dihydroquercetin substrate from the 1GP5 crystal structure and the structurally conserved residues TYR 151, ASN 237 and PHE 309 as guides. In this process, interactions with the side chains of TYR 151 and ASN 237 were used to anchor the thiocarboxyl oxygen and para-hydroxyl groups, respectively of the substrate while PHE 309 stabilized the substrate via π stacking with the ferrul ring. This arrangement places ARG 214 in position to interact with the oxygen lone pairs of the ferulic acid methoxy group, while residues SER 153 and ASN 216 are in position (slight side chain movements) to hydrogen bond with the thiocarboxyl oxygen.

### Structural Comparison of F6’H1 and C2’H Structures

**Figure 3:**
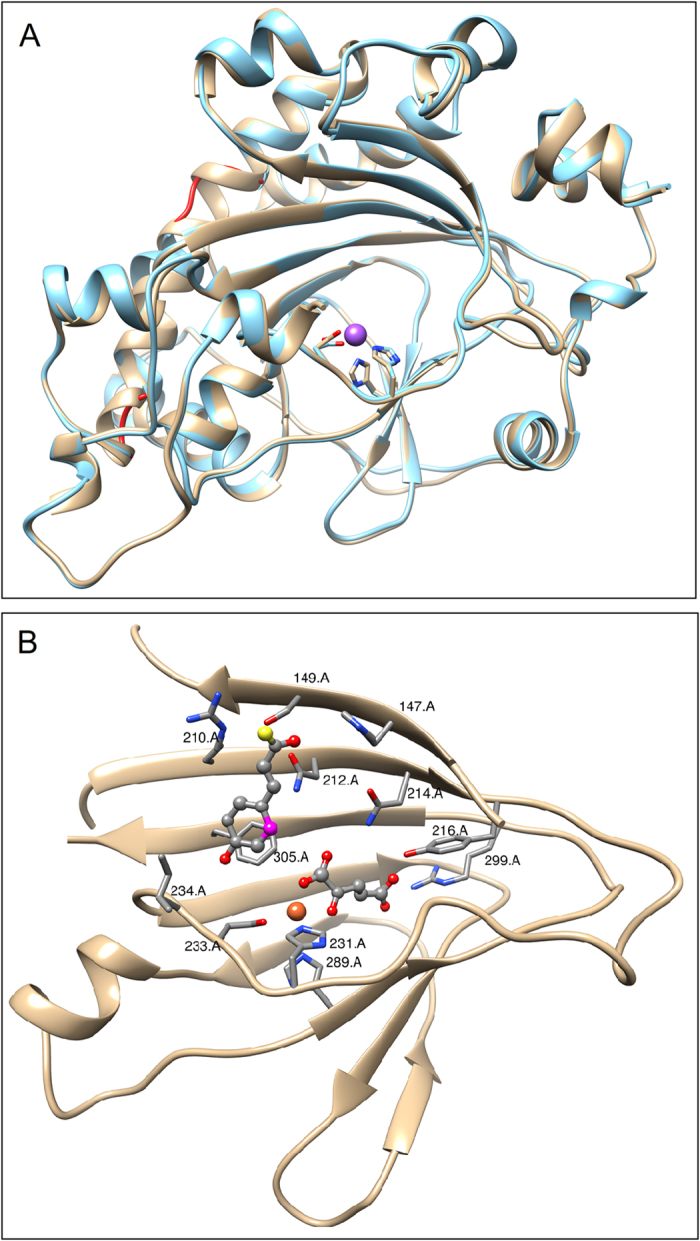
(**A**) A ribbon drawing of the C2’H homology model colored (cyan) superimposed on the F6’H1 crystal structure (tan). The sodium ion occupying the iron binding site is colored magenta. Regions showing the greatest structural deviations from the F6’H1 crystal structure are highlighted in red. (**B**) A ribbon drawing of a theoretical model of the C2’H 2OG-FeII_OXY domain showing active site components. The active site iron is colored orange. The 4-coumaroyl group of the 4-coumaroyl–CoA substrate (upper left) and 2OG (lower right) are depicted using ball and stick representations. Position C2 of the 4-coumaroyl group, the site of enzymatic attack is highlighted in magenta.

The C2’H homology model closely resembles the F6’H1 template used in the modeling. The two structures can be superimposed^16^ to give an RMSD of superposition of 0.407 Å for 320 α carbon pairs (Fig. 3A). The largest deviations between the two structures are observed for C2’H residues 174-177 (LKSC) which disrupts helix α 9. The sequence for the corresponding residues in the F6’H1 structure (residues 179 to 182) is NKSK.

The binding pocket in the C2’H homology model is also hydrophobic but is slightly larger at 2,938 Å^3^. A comparison of residues comprising the active site pocket (Fig. 3B) for the two enzymes shows that 86 of the 113 residues are structurally conserved including key residues involved in iron (HIS 231, ASP 233 and HIS 289), 2OG (ASN 216, TYR 220, ARG 299 and SER 301) and substrate (PHE 305) binding. However, in the C2’H structure TYR 151 which anchors the substrates thiocarboxyl oxygen in the active site has been replaced by HIS 147 and VAL 238 which sits at the edge of the pocket in close proximity to the substrate’s hydroxyl-benzyl group has been replaced by ILE 234. The remaining residues generally lie on the backside of the β strands with their side chains facing away from the active site or are located some distance from the catalytic center.

### Structural Features for Substrate Specificity

As noted above, the active sites of the two enzymes are structurally very similar and many of the enzyme substrate interactions are maintained in the two enzymes. The feruloyl-CoA and 4-coumaroyl-CoA substrates themselves are also very similar differing only by the presence of a methoxy group at the C4 position in feruloyl-CoA. The notable differences in enzyme substrate interactions are TYR 151 → HIS 147 and VAL 238 → ILE 234 substitutions, which may in part explain the substrate specificity of the two enzymes. Our mutation studies seem to support this idea (see below). The VAL 238 → ILE 234 replacement in C2’H would place a more bulky hydrophobic residue in the close proximity to the feruloyl-CoA methoxy group, which could sterically impact substrate binding or prevent the optimal placement of substrate for catalysis. The impact of the TYR 151 → HIS 147 replacement on substrate specificity is more difficult to explain structurally since both substrates have a thiocarboxyl oxygen at this position. The crystal structures of both active enzymes containing all active site components including the full substrate containing the CoA group should shed important new information on substrate specificity.

**Figure 4:**
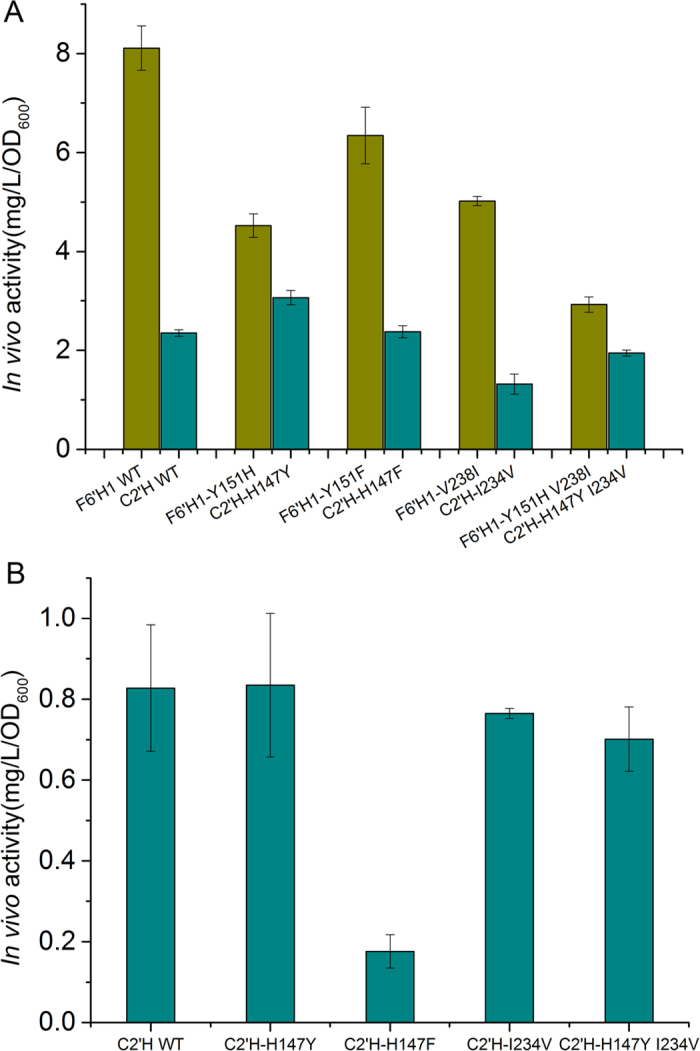
*In vivo* assays of F6’H1 and C2’H variants. (**A**) *In vivo* activity of F6’H1 variants and C2’H variants towards feruloyl-CoA and (**B**) *In vivo* activity of C2’H variants towards 4-coumaroyl-CoA. Y, TYR; H, HIS; F, PHE; V, VAL; I, ILE. Experiments were carried out in triplicate.

Site-directed mutagenesis was carried out to verify the roles of the above described amino acid residues on enzyme activity and specificity. For F6’H1 we introduced three single mutations (TYR151HIS, TYR151PHE and VAL238ILE) and a double mutation (TYR151HIS, VAL238ILE). Similarly, three single mutations (HIS147TYR, HIS147PHE and ILE234VAL) and one double mutation (HIS147TYR, ILE234VAL) were introduced into C2’H. As shown in Fig. 4A, the activity of all the F6’H1 mutants towards feruloyl-CoA was decreased compared with the wild type F6’H1 (F6’H1 WT), while C2’H HIS147TYR showed improved activity towards feruloyl-CoA. These results partly explain the higher activity of F6’H1 towards feruloyl-CoA than C2’H.

Interestingly, F6’H1 TYR151PHE and C2’H HIS147PHE mutants still showed relatively high activity towards feruloyl-CoA (Fig. 4A). On the contrary, compared with that of the wild type C2’H, the activity of C2’H HIS147PHE towards 4-coumaroyl-CoA decreased significantly (Fig. 4B). These results indicate that the interaction between TYR/HIS residues and the substrate thiocarboxyl oxygen was critical for hydroxylation of 4-coumaroyl-CoA but not for hydroxylation of feruloyl-CoA.

In addition, the activity of F6’H1 towards 4-coumaroyl-CoA was not improved by introducing those mutations, indicating that besides TYR 151 and VAL 238, the subtle difference between the overall structures of F6’H1 and C2’H is also important for the catalytic activity and substrate specificity. In the future studies, we plan to determine the crystal structure of C2’H to better understand the mechanisms of these important enzymes.

## Methods

### Expression and Purification of F6’H1

F6’H1 (GenBank Accession Number NP187970) cDNA was purchased from Arabidopsis Biological Resource Center (ABRC). The expression plasmid pETDuet1-F6’H1 was constructed by inserting F6’H1 gene into the BamHI/NdeI restriction sites of a pETDuet1 vector. *E. coli* strain BL21 Star (DE3) was then transformed with plasmid pETDuet1-F6’H1 containing an N-terminal 6XHis-tag (GSSHHHHHHSQD) to aid in purification. A fresh colony was inoculated into 50 mL of LB medium containing 100 μg/mL ampicillin and grown aerobically at 37 ^o^C overnight. The whole overnight culture was then used to inoculate 1 L of LB medium supplemented with 100 μg/mL ampicillin and grown at 37 ^o^C with shaking (250 rpm). When OD_600_ reached around 0.6, the culture was induced with 0.25 mM IPTG and cultivated at 30 ^o^C for an additional 3 hours.

Selenomethionine-substituted F6’H1 (Se-F6’H1) was produced using a metabolic inhibition protocol^26^. Briefly, 2 mL of cells from an overnight culture grown in LB medium containing 100 μg/mL ampicillin were collected by centrifugation and resuspended in 50 mL of M9 minimal medium containing 100 μg/mL ampicillin, 0.4% glucose, 2 mM MgSO_4_, vitamins, and trace elements. The culture was allowed to grow overnight and used to inoculate 4 × 1 L using the minimal media cocktail described above. The large-scale cell culture was shaken at 37 ^o^C until the OD_600_ reached around 0.6, at which point 100 mg of lysine, threonine and phenylalanine, and 50 mg of selenomethionine, leucine, isoleucine, and valine were added as solids into each liter of culture. Cells were then allowed to grow for an additional 20 min before IPTG was added to the final concentration of 1 mM. The resulting culture was grown overnight at 30 ^o^C.

For both the native and selenomethionine-substituted protein, the cells were harvested by centrifugation at 6000** × ***g* for 15 min at 4 ^o^C. The cell pellet was then suspended in 30 mL lysis buffer (20 mM phosphate buffer, pH 7.4, 500 mM NaCl, 20 mM imidazole, 10 ug/mL phenylmethylsulfonyl fuoride (PMSF)). The cell suspension was lysed by sonication on ice and cleared by centrifugation at 25,000** × ***g* for 30 min. The supernatant was then loaded onto a HisTrap HP column (5 mL, GE Healthcare) connected to AKTAprime plus (GE Healthcare) and pre-equilibrated with binding buffer (20 mM phosphate buffer, pH 7.4, 500 mM NaCl, 20 mM imidazole). The column was washed with 50 mL of binding buffer and the F6’H1 proteins eluted with a linear (20 to 500 mM) imidazole concentration gradient. The resulting purified proteins were dialyzed against 20 mM Tris-HCl, pH 7.4 containing 50 mM NaCl, 1 mM DTT and concentrated to approximately 12 mg/mL for crystallization.

### Crystallization, X-ray Data Collection and Structure Determination

Crystals of Se-F6’H1 were grown by sitting drop vapor diffusion at 291K using 2 μL drops containing equal volumes of protein concentrate and a precipitant cocktail containing 20% (w/v) PEG-8000, 0.1 M MES, 0.3 M Ca(OAc)_2_, pH 6.0. Crystals appeared in ~3 days and grew to usable size in 9-10 days.

For data collection a crystal measuring 200 × 200 × 50 microns was harvested from the well, briefly immersed in a drop of cryoprotectant solution containing the above precipitant cocktail with 20% (v/v) glycerol. The cryoprotected crystal was then flash cooled^27^ in liquid nitrogen and stored at cryogenic temperatures for data collection. A data set to 2.7 Å resolution was collected at 100 K on beamline 22ID, SER-CAT, Advanced Photon Source, Argonne National Laboratory using a 50 micron beam, a MAR300 CCD detector and 0.979 Å X-rays. A total of 360 one-degree images were recorded using a crystal-to-detector distance of 380 mm and an exposure time of 1 second. The data were indexed, integrated and scaled using HKL-2000^28^.

Initial attempts to solve the structure using SelenoMet SAD (single wavelength anomalous scattering)^29^ were unsuccessful and the structure was determined by molecular replacement (MR) using PHENIX^30^. The structure of Anthocyanidin synthase (ANS) from *A. thaliana* (PDB entry 1GP4)^24^, the closest PDB sequence homologue (34% identity), was used as the search model. Phaser-MR gave single molecular replacement solution containing a dimer in the asymmetric unit. Using this solution and two rounds of AutoBuild, the second employing noncrystallographic symmetry, 647 residues out of 746 (including His-tag) were built giving a map-model correlation of 0.78 and initial R and R_free_ values of 0.23 and 0.31, respectively. The model was further improved using iterative rounds of validation^31^, model building^32^ and refinement (using torsion angle noncrystallographic symmetry restraints). Since SelenoMet SAD was unsuccessful, the occupancies for the 6 selenium atoms were also refined. During the latter stages of refinement solvent molecules, modelled as water, were added to the model based on their environment and hydrogen-bonding scheme. Density was also observed at the iron-binding site and was modelled as a sodium ion since energy dispersive fluorescence scans of the crystal did not indicate the presence of iron. As outlined in Table 2 the refinement converged to give R and Rfree values of 0.2428 and 0.2999, respectively and had good stereochemistry, with RMSDs from ideality of 0.006 Å for bond lengths and 1.194° for bond angles. The coordinates and structure factors have been deposited in the Protein Data Bank as entry 4XAE.

### Homology Modelling of C2’H and Substrate Docking

A homology model of the C2’H structure was generated using Swiss Model V 3.70^33^ and the refined F6’H1 structure (63.3% sequence identity) as the template.

Models of the active site for the F6’H1 and C2’H enzymes Fe^2+^, 2-oxoglutarate (2OG) and the feruloyl component of feruloyl-CoA (F6’H1) and 4-coumaric acid component of 4-coumaroyl-CoA (C2’H) were visually positioned in the active site using CHIMERA^16^ with the crystal structures of Anthocyanidin synthase (PDB entries 1GP4, 1GP5, 1GP6 and 2BRT) serving as templates^24^,^34^.

### In vivo Assay of F6’Hs and C2’Hs

A coupled enzyme assay was used to evaluate the relative activity of F6’H1 and C2’H enzymes used in the analysis. Plasmids pZE-F6’H1-Pc4CL2 and pZE-C2’H-Pc4CL2 were constructed in our previous study^14^. Other plasmids were constructed by replacing F6’H1 encoding gene or C2’H encoding gene with their corresponding mutated genes. *E. coli* strain BW25113 was transformed with these plasmids, respectively. Overnight cultures were inoculated into 20 mL of M9Y media containing 100 μg/L ampicillin. Cell cultures were grown at 37 ^o^C with shaking. When OD_600_ reached 0.4, cells were induced with 0.25 mM IPTG for 3 h, at which time point the substrates ferulic acid or coumaric acid were fed into the cell cultures. After another 15 hours of incubation, samples were taken for OD_600_ measurement and supernatants after centrifugation were used for HPLC analysis. The enzyme activity was calculated based on the formation of the corresponding product (scopoletin or umbelliferone) and was expressed as mg/L/OD_600_. The ingredients of M9Y media, and the HPLC analysis method were described in our previous study^14^.

## Author Contributions

X.S., D.Z. and H.Z. expressed, purified and crystallized the protein. P.K. carried out the data collection, structure determination and refinement of the protein. X.S. carried out the mutational and enzyme activity analyses. X.S., D.Z. and J.R. wrote the manuscript. Q.Y., B.W., J.R. and Y.Y. supervised the project and/or commented on the manuscript.

## Additional Information

**How to cite this article**: Sun, X. *et al.* Structural Insights into Substrate Specificity of Feruloyl-CoA 6'-Hydroxylase from *Arabidopsis thaliana*. *Sci. Rep.* **5**, 10355; doi: 10.1038/srep10355 (2015).

## Figures and Tables

**Figure 1:**
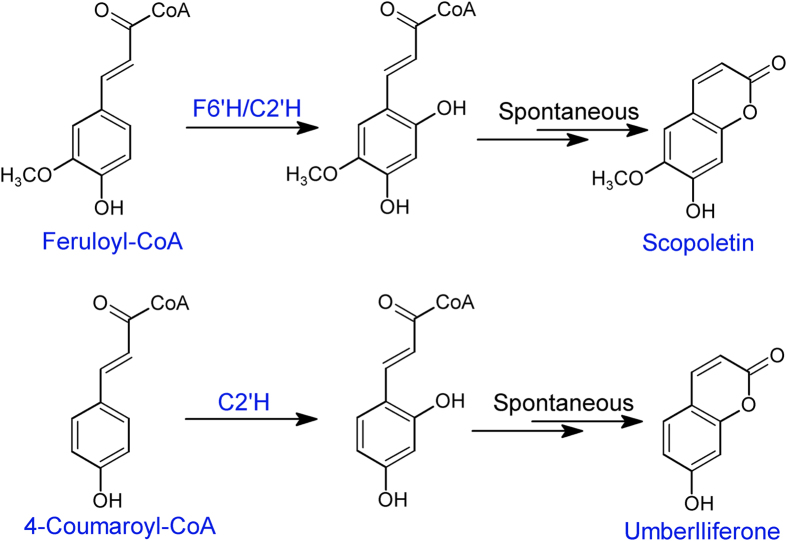
Representative of reactions catalyzed by F6’H1 and C2’H.

**Table 1:** Secondary structure assignments^35^.

α Helices	β Sheets
Helix α1 VAL 16 VAL 21	Sheet S1 β1 VAL 64 ILE 65
Helix α2 ASN 25 GLU 32	Sheet S1 β2 PHE 88 VAL 91
Helix α3 GLU 40 TYR 42	Sheet S1 β10 ILE 267 ILE 271
Helix α4 LEU 46 ASN 52	Sheet S1 β7 LEU 238 HIS 243
Helix α5 GLU 72 TRP 86	Sheet S2 β12 ARG 298 GLU 306
Helix α6 LEU 98 ASN 113	Sheet S1 β5 LEU 209 TYR 217
Helix α7 VAL 116 LYS 119	Sheet S1 β4 ASP 141 TYR 148
Helix α8 ARG 120 PHE 122	Sheet S1 β3 GLY 124 GLY 126
Helix α9 GLU 159 GLN 164	
Helix α10 ARG 171 TYR 191	Sheet S2 β6 VAL 229 HIS 232
Helix α11 LYS 194 LEU 196	Sheet S2 β11 HIS 288 GLY 290
Helix α12 THR 204 MSE 210	Sheet S2 β8 LEU 249 TYR 253
Helix α13 ASP 278 SER 284	Sheet S2 β9 LYS 253 THR 259
Helix α14 PRO 322 VAL 324	
Helix α15 TYR 338 VAL 342	Sheet S3 β13 VAL 313 LEU 314
	Sheet S3 β14 ARG 332 THR 334

**Table 2:** Crystallographic data and refinement statistics.

**Data collection**	
Diffraction source	APS 22-ID
Wavelength (Å)	0.979
Temperature (K)	100
Detector	MAR300
Rotation range per image (°)	1
Total rotation range (°)	360
Exposure time (sec)	1
**Crystal data**	
Space group	C2
Unit-cell parameters	
a (Å)	193.22
b (Å)	54.55
c (Å)	78.82
γ(°)	111.5
Resolution (Å)	44.93-2.70 (2.795-2.698)
Total reflections	557846
Unique reflections	19697 (1250)
Completeness (%)	92.21 (59.50)
Multiplicity	6.2 (3.2)
R_merge_	0.139 (0.366)
R_meas_	0.127 (0.414)
R_p.i.m_	0.49 (0.199)
< I/σI>	6.70 (2.01)
CC_1/2_	87.4
CC*	96.6
Matthews coefficient (Å^3^ Da^−1^)	2.38
**Refinement statistics**	
Reflections, working set	18707
Reflections, test set	1946
Resolution range (Å)	46.703- 2.769
Completeness (%)	94.43
R factor	0.2428
Rfree	0.2999
No. of non-H atoms	
Protein	5141
Ligand	2
Water	28
R.m.s. deviation from ideal	
Bond lengths (Å)	0.006
Bond angles (°)	1.194
Ramachandran plot† (%)	
Residues in favoured region	94.25
Residues in allowed region	4.04
Outliers	1.71
MolProbity score	8.81
Poor rotamers‡ (%)	1.06
PDB code	4XAE

Statistics for the highest-resolution shell are shown in parentheses. *R*_merge_ = ∑_*hkl*_ ∑_*i*_|**I**_*i*_ (*hkl*) – 〈*I*(*hkl*)〉|/∑_*hkl*_ ∑_*i*_ *I*_*i*_(*hkl*), where *I*_*i*_ (*hkl*) is the observed intensity and <*I*(*hkl*)> is the average intensity over symsmetry-equivalent measurements. *R*_r.i.m._ = ∑_*hkl*_ {*N*(*hkl*)/[*N*(*hkl*) –1]}^1/2^ ∑_*i*_|*I*_*i*_(*hkl*)–〈*I*(*hkl*)〉|/∑_*hkl*_ ∑_*i*_ *I*_*i*_(*hkl*). *R*_p.i.m._ = ∑_*hkl*_ {1/[*N*(*hkl*)–1]}^1/2^ ∑_*i*_|*I*_*i*_ (*hkl*) – 〈*I*(*hkl*)〉|/∑_*hkl*_ ∑_*i*_ *I*_*i*_ (*hkl*). *Rfactor* = ∑_*hkl*_ │|*F*_obs_| – |*F*_calc_|│/∑_*hkl*_ |*F*_obs_|, where *R*_free_ is calculated for a random chosen 5% of reflections which were not used for structure refinement and *Rfactor* is calculated for the remaining reflections.
